# British Laryngological, Rhinological, and Otological Association

**Published:** 1897-11-20

**Authors:** 


### BRITISH LARYNGOLOGICAL, RHINOLOGICAL, AND OTOLOGICAL ASSOCIATION.

At the last meeting of this society several points of considerable interest were raised. The President, Dr. Dundas Grant, showed a series of cases of frontal headache of nasal origin. In one case it seemed to depend on disease in the frontal sinus, in another on disease in the sphenoidal sinus, and in a third on suppuration in the antrum of Highmore. It was pointed out in the discussion that the possibility of frontal headache having its origin in such conditions was a matter of some importance, and a thing to be borne in mind, while the cases served to indicate again the fact that hard and fast lines as to diagnosis of the particular cavity attacked, by the localisation of the pain, were not to be relied upon. A communication from Mr. Tressilian as to four cases of enteric fever which exhibited throat lesions, out of eight examined within the last two months, served to raise a discussion as to the frequency of throat lesions in this disease. Mr. Lennox Browne thought it regrettable that the throat and the tympanic membrane were rarely looked at in enteric fever, and suggested that the fact that throat trouble was the rule rather than the exception in that disease, pointed to the desirability of carrying specialism a little more into the fever hospitals. The President drew attention to the terrible cases of laryngeal stenosis resulting from perichondritis in enteric fever, which he thought accentuated the necessity for more care being taken of the throat and mouth during the progress of this disease.

